# Associations between Perceived HIV Stigma and Quality of Life at the Dyadic Lvel: The Actor-Partner Interdependence Model

**DOI:** 10.1371/journal.pone.0055680

**Published:** 2013-02-01

**Authors:** Hongjie Liu, Yongfang Xu, Xinjin Lin, Jian Shi, Shiyi Chen

**Affiliations:** 1 Department of Epidemiology and Biostatistics, School of Public Health, University of Maryland, College Park, Maryland, United States of America; 2 Nanning Center for Disease Control and Prevention, Nanning, Guangxi, China; Rollins School of Public Health, Emory University, United States of America

## Abstract

**Background:**

Few studies have investigated the relationship between HIV-related stigma and quality life at the dyadic level. The objective of this study was to examine the actor and partner effects of stigma that was perceived by people living with HIV/AIDS (PLWHAs) and caregivers on quality of life at the dyadic level.

**Method:**

A survey was conducted among 148 dyads consisting of one PLWHA and one caregiver (296 participants) in Nanning, China. The interdependent relationship between a pair of dyadic members that influences the associations between stigma and quality of life was analyzed, using an innovative dyadic analysis technique: the Actor-Partner Interdependence Model (APIM).

**Results:**

We found in this dyadic analysis that (1) PLWHAs compared to their caregivers exhibited a higher level of perceived HIV stigma and lower level of quality of life measured in four domains; (2) both PLWHAs' and caregivers' perceived HIV stigma influenced their own quality of life; (3) The quality of life was not substantially influenced by their partners' perceived stigma; and (4) Both actor and partner effects of stigma on quality of life were similar among PLWHAs and their caregivers.

**Conclusion:**

As HIV stigma and quality of life are complex phenomena rooted in cultures, intervention programs should be carefully planned based on social or cognitive theories and should be culturally adopted.

## Introduction

The number of people living with HIV/AIDS (PLWHAs) continues to increase in China. Data from the China National HIV Surveillance System indicates that newly-diagnosed HIV cases have been increasing yearly from 2007 to 2011 (e.g., 10,742 to 39,183 cases) [1]. Sexual contacts and injection drug use are the primary modes of HIV transmission. By the end of 2011, the cumulated number of PLWHAs was 780,000 [1]. Twenty percent of PLWHAs progressed to AIDS cases (full-blown AIDS). China's population is aging fast, as are PLWHAs. The proportion of PLWHAs who were aged 50 years or older rose dramatically from 1.9% in 2000 to 21.1% in 2011, an 11-fold increase [2]. Because of the increased number of PLWHAs and frequency of HIV-related chronic disease conditions, issues regarding quality of life in these individuals have emerged in China.

The World Health Organization (WHO) defines quality of life as ‘individuals' perception of their position in life in the context of the culture and value systems in which they live and in relation to their goals, expectations, standards and concerns.’ [3] This definition indicates that quality of life refers to a subjective assessment which is embedded in a cultural, social and environmental context [4]. Because HIV/AIDS is a chronic disease, PLWHAs suffer a variety of HIV-associated co-morbid conditions, and psychosocial challenges which may ultimately impact quality of their life. Moreover, these conditions may influence quality of life of PLWHAs' close associates, for example, spouses, parents, children, or those who provide care to them. The co-occurrence of impaired quality of life in PLWHAs and their close associates may be particularly prevalent in the Chinese collectivist culture. Individuals in this collectivist society are advised to exercise emotional restraint in order to avoid shame and “save face” for their whole families [5]. The cultural imperative of familial responsibility, rather than individual rights, may foster perception of impaired quality of life in both PLWHAs and their family members. Although research has investigated quality of life and its determinants among PLWHAs, quality of life of their caregivers has long been ignored, especially, its potential psychosocial determinants. Studies of other chronic diseases have found that quality of life of caregivers of individuals with affective disorders (depressive disorder or bipolar disorder) and schizophrenia was seriously impaired [6], [7], [8]. As HIV stigma prevails among PLWHAs and their caregivers, it may influence mental health and quality of life among both PLAHAs and their caregivers [9], [10].

Stigma has been described as a quality that “significantly discredits” an individual in the eyes of others [11]. It is socially constructed and reinforced by social inequality [12]. Members of collectivist cultures subordinate individual interests to that of the group or collective [13], [14]. As a result, Chinese pay more attention than individuals in other cultures to how they would be evaluated or viewed by others. It has been reported that the Chinese society displays an unusually high degree of stigma and stigma associated with mental illness was more severe than in the West [15]. As posited by Goffman [16], stigma can be passed on to family members of those with the stigmatizing attribute, terming this phenomenon “courtesy stigma”. Courtesy stigma refers to the stigma that attaches to those who are merely associated with a stigmatized person (e.g., PLWHA). The importance of saving family face and family dignity ensures that not only the HIV infected persons, but also their family members are highly stigmatized in China [17], [18]. Stigmatized caregivers often suffer physical and mental health problems [19], [20], [21], which may lead to impaired quality of life. Research has documented that HIV-related stigma has a negative impact upon quality of life in PLWHAs [22]. However, the relationship between quality of life and stigma is not clear among caregivers of PLWHAs. As stigma and impaired quality of life take place among both PLWHAs and their caregivers, the relationship between stigma and quality of life is potentially interwoven or interdependent between PLWHAs and their caregivers at the dyadic level, leading to research questions, such as, “Was PLWHAs' quality of life not only determined by their own perception of HIV stigma, but also their caregivers'?”

The anticipated interdependence relationship may be explained or supported by the Theory of Interdependence [23], [24]. Central to the theory is the idea that dyadic partners affect each other and that the partnership impacts both individual's lives. According to this theory [24], interdependence refers to the manner in which – as well as the degree to which – interacting individuals act upon or influence one another's experiences or lives. The theory of Interdependence promotes the examination of interaction and relationships by delineating the ways in which psychosocial situations shape both intrapersonal and interpersonal processes [25].

However, the ability to investigate how dyadic situations shape both intrapersonal and interpersonal processes has been hampered by problems that arise when treating the individual, rather than the dyad, as the unit of analysis. The dyad - whether a marital relationship, a friendship, a kinship, or even the relationship between PLWHAs and their caregivers, is the fundamental unit of interpersonal relations and interaction. Through interpersonal interactions, members in the interdependent relationships made a distinct contribution to developmental outcomes of their own and their partners' in the forms of cognitions, emotions, and behaviors [26], [27]. Conventional parametric statistics (e.g., t-test, χ^2^ test, analyses of variance, and linear or logistic regression models) are built upon the assumption of independence of observations. When the assumption of independence is violated, the test statistics are inaccurate, and their statistical significance is biased. Clearly, interdependence within interpersonal relationships in dyads violates the independence assumption. However, according to the theory of interdependence, it is the interdependent relationship between a pair of dyadic members that influences the emotion, cognition, or behavior of each of them. Specific statistical methods need to be used to examine and test the interdependent relationship and its consequences.

Kenny and colleagues have developed a model of dyadic data analysis, the Actor-Partner Interdependence Model (APIM) [26]. The APIM uses the dyad as the unit of analysis and provides separate but simultaneous estimates of actor and partner effects. An actor effect occurs when a person's score of a predicting variable affects that person's score of an outcome variable. A partner effect represents when a person's score of a predicting variable affects the score of an outcome of that person's partner. For example, the effect of a PLWHA's perceived stigma (the predictor) on his/her own quality of life (the outcome) is the actor effect, and the effect of that PLWHA's perceived stigma on his/her caregiver's quality of life is the partner effect. In addition, the APIM can be used to impose equality constraints to test specific hypotheses regarding various effects, which allows us to answer questions such as, “Is the actor effect of perceived stigma on quality of life the same for both PLWHAs and their caregivers?”.

The objective of this study is twofold: to contribute to the substantive literature on quality of life and stigma by examining their differences between PLWHAs and their caregivers, and to use the APIM to elucidate and differentiate actor effects and partner effects of perceived stigma on quality of life. We examine the following research questions: (1) Is an individual's level of quality of life associated with his/her own level of perceived HIV stigma (actor effect)?; (2) Is an individual's level of quality of life associated with his/her partner's level of stigma (partner effect)?; and (3) Does the actor effect of perceived stigma on quality of life differ significantly between PLWHAs and their caregivers?”

## Materials and Methods

The study protocol and consent procedures were reviewed and approved by the Institutional Review Boards of Virginia Commonwealth University and Guangxi Center for Disease Control and Prevention. In accordance with the approved protocol, written informed consent was obtained from all study participants prior to data collection.

### Study sites and subjects

This cross-sectional study was conducted in Nanning, the capital city of Guangxi province. Guangxi is located along a drug trafficking route, which originates in the “Golden Triangle”, passes through northern provinces of Vietnam and finally leads into Hong Kong and the rest of the world [28]. Guangxi ranks second among China's 31 provinces in terms of the estimated number of PLWHAs in 2011. The major HIV transmission routes are needle sharing and heterosexual sex [1].

We purposely selected three study sites in the city that provided HIV care and treatment services for the majority of PLWHAs: an infectious disease hospital that was designated to provide care and treatment for PLWHAs, a methadone maintenance treatment (MMT) clinic run by Nanning Center for Disease and Control, and a health-care center run by PLWHA volunteers. Eligibility criteria for PLWHAs included PLWHAs who were at least 18 years old and able to participate in a face-to-face interview. Based on the list of PLWHAs in each site, we first invited and interviewed eligible PLWHAs at the study sites. We then invited their caregivers to go to the sites and receive a face-to-face interview in a private room. The criteria for the selection of caregivers included individuals who: (1) were the primary caregivers to the index PLWHAs, (2) at least 18 years old, and (3) HIV negative. Of 170 HIV dyads invited to participate in this study, 20 dyads refused, and 2 dyads did not provide information about their perceived stigma and thus were excluded, resulting in a total of 148 dyads in data analysis.

### Measures

Measurement items and scales were initially drafted in English and then translated into Chinese by three research members who were fluent in both languages. The Chinese version of the measurement items was then distributed to research team members who reviewed and modified the wording to make it appropriate for the Chinese context.

Quality of life: The Chinese version of the World Health Organization Quality of Life Assessment (WHOQOL-BREF) was used to measure the level of quality of life in both PLWHAs and their caregivers [29], [30]. Its psychometric properties were assessed in a survey of adults carried out in 23 countries, including China [29]. The WHOQOL-BREF consists of 26 items that measure four QOL-domains: physical health (pain, energy, sleep, mobility, activities, medication, work), psychological domain (positive and negative feelings, cognitions, self-esteem, body image, spirituality), social relationships (personal relationships, social support, sexual activities) and environmental aspects (safety and security, home environment, finances, health and care, information, leisure, physical environment, transport). Interviewees respond to these items on a five-point Likert scale. The four domain scores indicate an individual's perception of quality of life in each particular domain. Domain scores are scaled in a positive direction (i.e., higher scores indicate higher quality of life). The reliability of the measurement domains was between 0.60 and 0.83. A mean score of items within each domain was used to calculate the domain score. Mean scores were then multiplied by 4 in order to make the four individual domain scores comparable in the four domains [31].

Perceived HIV stigma: Perceived or felt stigma refers to an individual's anticipated fear of societal attitudes and potential discrimination if they were to have a particular undesirable attribute, such as HIV infection [32], [33]. Based on our experience in design of stigma measurement scales [34], [35], [36], we designed a 9-item measurement scale to measure the level of perceived HIV stigma among both PLWHAs and their caregivers (e.g., *I feel that my neighbors or co-workers would avoid me if they knew I was HIV positive or if they knew there was an HIV case in my family; I feel that others would look down up me if they knew I was HIV positive or if they knew there was an HIV infected member in my family*). Participants responded on a four-point scale ranging from “strongly disagree (0)” to “strongly agree (3).” Cronbach's alpha was 0.89 in PLWHAs and 0.92 in their caregivers. The composite score ranged from 0 to 27.

### Data analysis

In the descriptive analysis, paired-sample *t* test and McNemar χ^2^ test were used to determine statistical differences in demographic variables (age, gender, marital status, and education) between PLWHAs and caregivers. Pearson product-moment correlation coefficients were estimated to examine correlations among stigma and the four domains of quality of life.

**Figure 1:**
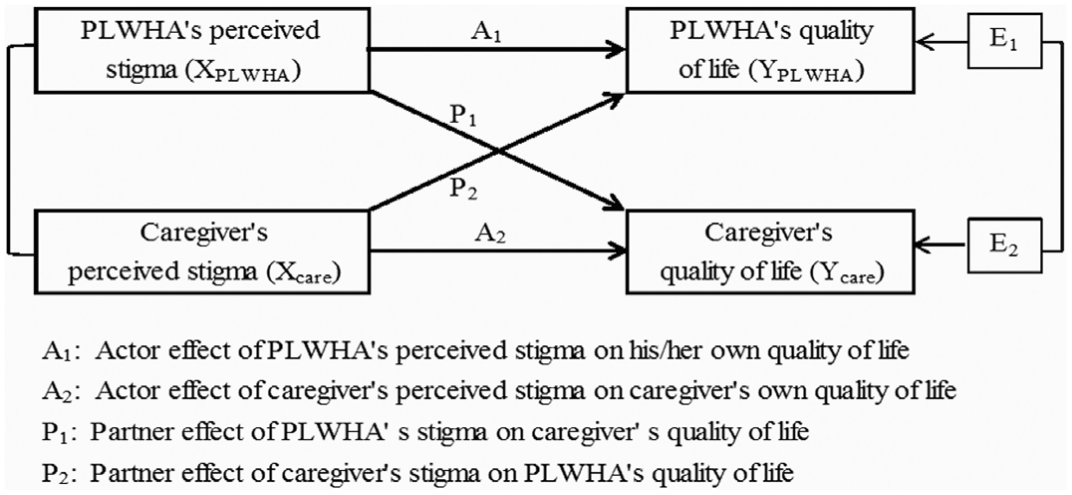
Actor Partner Interdependence Model Examining Actor and Partner Effects on Quality of Life.

To determine the impact of PLWHAs and caregivers' stigma on their own quality of life as well as their partners' quality of life, the APIM with distinguishable dyads was performed [26]. Figure 1 depicts the APIM of a dyad in which there is one PLWHA and one caregiver, and two variables measured from each in the dyad: perceived HIV stigma (independent variable) and quality of life (outcome variable). The PLWHA's level of quality of life is influenced by his/her own level of perceived stigma (actor effect, A_1_) and by his/her caregiver's stigma level (partner effect, P_1_) as well. Similarly, the caregiver's level of quality of life is affected by his/her own perception of stigma (actor effect, A_2_) and the PLWHA's stigma level (partner effect, P_2_). There are two correlations in this model, the correction of the PLWHA's stigma score and the caregiver', and the correlation of two residual non-independences in the outcome scores (E_1_ and E_2_), which represents the non-independence that is not explained by the APIM.

Three statistic modeling techniques can be used to estimate the APIM: pooled regression modeling, multilevel modeling, and structural equation modeling. According to Kenny and colleagues [26], structural equation modeling (SEM) with distinguishable dyads is the simplest and most straightforward analytic method for estimating the APIM. The SEM approach involves estimating the APIM parameters as they appear in the model presented in Figure 1. Based on the dyad-level structure, two linear equations are written here:
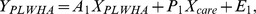


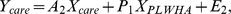
where *Y_PLWHA_* is the score of PLWHA's quality of life, *Y_care_* is the score of caregiver's quality life, *X_PLWHA_* is the score of PLWHA's perceived HIV stigma, and *X_care_* is the score of caregiver' s stigma. In the first equation, *A_1_* is the regression coefficient measuring the actor effect of PLWHA's stigma on his/her own quality of life, and *P_1_* measures the partner effect of caregiver's stigma on the PLWHA's quality of life. Similarly, in the second equation, *A_2_* is the coefficient measuring the actor effect of a caregiver's stigma on his/her own quality of life, and *P_2_* is the partner effect of PLWHA's stigma on caregiver's quality of life. As the dyad is the unit of analysis, the sample size in this analysis is the number of dyads (not the total number of subjects). Goodness of fit in the APIM was assessed with the χ^2^/degree ratio (<2), the comparative fit index (CFI≥0.9), and the standardized root mean square residual (SRMR≤0.06).

A useful feature of SEM is that it uses the equality constraint test to statistically compare and evaluate the size of parameters within the model. For example, it can test whether the PLWHA actor effect is equal to the caregiver actor effect (two parameters), which answers the question of who has more influence in the relationship [37]. The equality constraint test compares the value of the chi-square test of model fit for a model with the two parameters that are constrained to be equal to the chi-square test of model fit for the same model but without the constraints. If the difference between the two chi-square values is statistically significant, then constraining the parameters to be equal has significantly worsened the fit of the model. Therefore, it is inferred that the parameters are not equal. To compute an equality constraint test, the difference of the chi-square values of the two models in question is taken as well as the difference of the degrees of freedom (df).
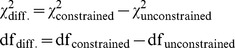



*SAS* (version 8) was used in descriptive analysis of the sample demographics. *Mplus* (version 7) was used to apply structural equation modeling in the APIM [38].

## Results

### Characteristics of dyads of PLWHAs and caregivers

Among 148 PLWHAs, 60% (89/148) were diagnosed with HIV or AIDS in the year prior to the interview, 28% (42) in the past two or three years, and 12% in the past four or more years; and 54% (80) received antiretroviral therapy (ART). Among 148 caregivers, 37% (55) were PLWHAs' spouse, 28% (42) were their brother or sister, 25% (37) were their parents, and 10% were other relationships (e.g., grandparents, brother in law, or sister in law). On average, PLWHAs were 3.2 years older than caregivers, but had a similar education level as their caregivers. Compared to PLWHAs, more caregivers were female and married (Table 1).

**Table 1:** Demographics, stigma and quality of life between PLWHAs^1^ and caregivers.

	PLWHAs	Caregivers	p - value
	No. (%)	No. (%)	
Gender			<0.01
Female	45 (30.4)	90 (60.8)	
Male	103 (69.6)	58 (39.2)	
Education			0.52
No school or primary school			
	38 (25.7)	34 (23.0)	
Middle school or above			
	110 (74.3)	114 (77.0)	
Marital status			0.04
Unmarried	44 (29.7)	30 (20.3)	
Married	104 (70.3)	118 (79.7)	
	Mean (SD)	Mean (SD)	
Age (years)	40.7 (11.4)	37.5 (11.2)	0.01
Perceived HIV stigma			
	6.3 (5.8)	3.8 (5.3)	<0.01
WHOQOL-BREF			
Physical health	11.8 (3.0)	14.5 (2.2)	<0.01
Psychological	11.1 (2.8)	13.1 (2.6)	<0.01
Social relationships	12.8 (2.6)	14.2 (2.5)	<0.01
Environment	10.8 (2.5)	11.9 (2.5)	<0.01

1 people living with HIV/AIDS.

### Perceived HIV stigma and quality of life in PLWHA-caregiver dyads

On average, the level of perceived HIV stigma among PLWHAs was 2.5 higher than that among caregivers. The levels of the four domains that measure the quality of life were statistically significantly lower among PLWHAs than those among their caregivers, indicating that PLWHAs' quality of life might be poorer than their caregivers' (Table 1).

PLWHAs' perception of HIV stigma was significantly and negatively correlated with their own four domains of quality of life: physical health (correlation coefficient = −0.29), psychological (correlation = −0.34), social relationship (correlation = −0.28), and environmental (correlation = −0.42). It was also negatively correlated with caregivers' two domains of quality of life, i.e., physical health (coefficient = −0.21) and social relationship (correlation = −0.21). However, it was not significantly correlated with their caregivers' stigma and two other domains of quality of life (psychological and environmental) (Table 2).

**Table 2:** Correlations among perceived HIV stigma and four domains of quality of life.

		Cronbach's alpha	2	3	4	5	6	7	8	9	10
People living with HIV/AIDS											
1	Perceived HIV stigma	0.89	−0.29**	−0.34**	−0.28**	−0.42**	0.15	−0.21**	−0.11	−0.21*	−0.11
2	Physical health	0.84		0.77**	0.53**	0.58**	−0.05	0.11	0.04	0.06	0.14
3	Psychological	0.83			0.55**	0.66*	0.02	0.09	0.13	0.10	0.17*
4	Social relationships	0.60				0.55**	−0.03	0.12	0.09	0.17*	0.09
5	Environment	0.84					−0.04	0.20*	0.17*	0.20*	0.29**
Caregivers											
6	Perceived HIV stigma	0.92						−0.42*	−0.50**	−0.36**	−0.40**
7	Physical health	0.74							0.75**	0.55**	0.61**
8	Psychological	0.79								0.57**	0.71**
9	Social relationships	0.63									0.59**
10	Environment	0.83									
	* p≤0.05 ** p≤0.01										

Caregivers' perception of HIV stigma was significantly and negatively correlated with their own four domains of quality of life: physical health (correlation coefficient = −0.42), psychological (correlation = −0.50), social relationship (correlation = −0.36), and environmental (correlation = −0.40). However, it was not significantly correlated with PLWHAs' perception of stigma and quality of life of PLWHAs.

### Impact of perceived HIV stigma on quality of life at the dyadic level

The results of the model fit test indicate that the four APIMs fit the data well (Table 3). As documented by the results of the APIM, perceived HIV stigma exerted statistically significant actor effects on quality of life measured in the four domains for both PLWHAs and their caregivers (Table 3). With regard to partner effects, however, there were no significant partner effects on quality of life for both PLWHAs and their caregivers. One exception is that the caregivers' social relationship domain was significantly associated with both their own stigma and their PLWHA partners'. However, the level of association was much higher in the actor effect (−0.13) than the partner effect (−0.06). These results indicate that PLWHAs and caregivers with higher levels of perceived HIV stigma might have poorer quality of life. The status of quality of life was mainly influenced by their own level of stigma, not by their partners'.

**Table 3:** Actor and partner effect of perceived HIV stigma on quality of life.

	PLWHA^1^s		Caregivers		Model fit		
	aβ^2^	95% CI^3^	aβ	95% CI	?^2^ ratio^4^	CFI^5^	SRMR^6^
Physical health					1.50	0.93	0.03
Actor's perceived stigma	−0.13	−0.21–−0.05**	−0.15	−0.21–−0.08**			
Partner's perceived stigma	−0.02	−0.10–0.06	−0.05	−0.11–0.003			
Psychological					1.36	0.97	0.03
Actor's perceived stigma	−0.15	−0.22–−0.07**	−0.21	−0.27–−0.14**			
Partner's perceived stigma	0.02	−0.07–0.10	−0.002	−0.06–0.06			
Social relationships					0.93	1.00	0.02
Actor's perceived stigma	−0.11	−0.18–−0.04**	−0.13	−0.21–−0.06**			
Partner's perceived stigma	−0.006	−0.09–0.07	−0.06	−0.13–−0.001*			
Environment					0.63	1.00	0.02
Actor's perceived stigma	−0.17	−0.23–−0.10**	−0.17	−0.24–−0.10**			
Partner's perceived stigma	0.005	−0.07–0.08	−0.02	−0.07–0.04			

1 people living with HIV/AIDS.

2 adjusted coefficient, adjusted for age, gender, marital status, and education.

3 95% confidence interval.

4 χ2/degree ratio.

5 Comparative fit index.

6 standardezed root means square residual.

The equality constraint tests indicated that the actor effects did not significantly differ for PLWHAs and caregivers (Table 4). That is, the actor effects of stigma on the quality of life were statistically similar for both PLWHAs and their caregivers. The same results were also seen in the partner effects.

**Table 4:** Equality constraint tests regarding model fit.

	?^1^_diff._	df _diff._ 2	p -value
Physical health			
Equated actor's perceived stigma	0.07	1	0.79
Equated partner's perceived stigma	0.48	1	0.49
Psychological			
Equated actor's perceived stigma	1.29	1	0.26
Equated partner's perceived stigma	0.12	1	0.73
Social relationships			
Equated actor's perceived stigma	0.19	1	0.66
Equated partner's perceived stigma	1.23	1	0.27
Environment			
Equated actor's perceived stigma	0.01	1	0.92
Equated partner's perceived stigma	0.16	1	0.69

1 difference of chi square test values.

2 difference of degrees of freedom.

## Discussion

In this dyadic analysis, we found that: (1) PLWHAs compared to their caregivers exhibited a higher level of perceived HIV stigma and poorer quality of life; (2) both PLWHAs' and caregivers' perceived HIV stigma influenced their own quality of life; (3) quality of life was not substantially influenced by their partners' perceived HIV stigma; and (4) both actor and partner effects of stigma on the quality of life were similar in PLWHAs and their caregivers.

As expected, PLWHAs compared with their caregivers possessed a higher level of perceived HIV stigma, but a lower level of quality of life. In a study of quality of life among PLWHAs in Henan, China [39], Shan and colleagues reported that the physical, psychological, social, and environmental domain scores were 12.9±1.95 (mean ± standard deviation), 12.4±1.80, 14.0±2.43, and 12.5±1.91, respectively. We used t-tests to test differences between the score of each domain in our study versus the score reported in theirs and found that the levels of the four domains in our study were significantly lower than their findings, indicating that the quality of life among PLWHAs in our study might be poorer than quality of life reported in their study. This difference could be due to the different stages of disease progression, effects of anti-retroviral treatment, or other psychosocial factors. In Henan province, the majority of PLWHAs acquired HIV through commercial blood donation, a less stigmatizing transmission mode compared to sexual or needle-sharing transmission modes in our study. Similarly, we compared caregivers' scores of the four domains with scores that were reported in a study of a sample of a general population in China [29], in which mean scores were15.8±2.9 in physical health, 14.3±2.5 in psychological domain, 13.7±3.0 in social relationships, and 13.2±2.4 in environmental domain. The scores of physical health, psychological domain, and environment domain among PLWHAs in our study were significant lower than the scores reported in the general population, but no significant difference was observed for the score of social relationship. Clearly, PLWHAs in our study had poorer quality of life, compared with their caregivers who in turn might have poorer quality of life compared with the general population.

Consistent with previous studies [22], [40], [41], our study found the actor effect of stigma on quality of life. In other words, the higher level of HIV stigma that is perceived by either PLWHAs or their caregivers contributes to lower quality of life for themselves. HIV stigma influences a person's willingness to seek social support, medical care and motivation to adhere to therapy. It also limits a person's self-esteem and efficacy in problem- or depression-focused coping, emotional or tangible exchanges within or across personal networks, and abilities in physical activities. As these consequences of stigma involve the influence of the four domains of quality of life, stigma ultimately affects a person's overall quality of life [41]. As documented in a recent study in China [40], HIV stigma mediates the relationship between self-efficacy, medication adherence, and quality of life among PLWHAs. Although caregivers' stigma level is much lower than PLWHAs, the actor effect of caregiver's stigma on their quality of life is exhibited in our study. In order to save ‘face’ or dignity of their family, caregivers usually do not disclosed the presence of HIV to others outside of their family. They may thus internalize HIV-related stigma [42]. Here, internalization of stigma is the anticipation of negative attitudes that would be experienced by the caregiver. Thus, these caregivers do not want others to have the opportunity to discriminate, devalue or ostracize their family members. They may display a behavioral response to potential negative attitudes toward HIV that requires psychosocial coping or problem management, which may eventually induce their perceived or actual loss in quality of life. As HIV/AIDS is a chronic disease that places stress on the entire family [9], psychological distress, such as stigma, depression and anxiety, has been found to impact all members of a family coping with the chronic disease [43], [44], [45].

One of the contributions of this study is that it is the first to examine the proposition that HIV perceived stigma of one member of the dyad is associated with quality of life of his or her partners. However, in contrast to our expectation, our study does not demonstrate a strong partner effect of HIV stigma on quality of life, although stigma perceived by PLWHAs is marginally associated with their caregivers' one domain of quality of life. One possible explanation is that stigmatizing attitudes or behaviors towards to PLWHAs or caregivers originates from outside the family, e.g., their network peers, neighbors, coworkers, or others in their community. That is, neither PLWHAs nor their caregivers possess such stigmatizing attitudes or discriminate towards each other within dyads. Therefore, a partner's perceived stigma does not contribute to an individual's quality of life beyond the individual's own perceived stigma. Another possible reason is that the PLWHA and his/her caregivers in a family help each other to cope with HIV stigma in order to fulfill their family responsibility. In China, the family forms an important safety umbrella for PLWHAs, and family members are the primary caregivers for care, treatment, psychosocial and financial support, and childcare. Family members usually obtain support from their family members rather than from outsiders. In this case, stigma perceived by one dyadic individual may not influence quality of life of the other in the dyad. Since there is little literature to compare our findings examining the partner effect of HIV stigma on quality of life, our hope is that these findings will contribute to this growing field.

This study documents that the actor effects and partner effects of stigma on the quality of life were similar for both PLWHAs and their caregivers. Although the levels of HIV perceived stigma and the four domains of quality of life differed between PLWHAs and caregivers, the associations between stigma and quality of life were not substantially different between the two groups. This result may indicate that the two groups share a similar mechanism through which HIV perceived stigma influences quality of their life.

Several limitations to this study should be noted. First, the single study site with a purposely chosen convenient sample may limit generalizability of our findings. Future large-scale studies are needed to confirm these findings. Second, due to the cross sectional nature of this study, these data should be interpreted as associations rather than implying causality. Third, this study relied on self-reported data and consequently may have some limitations because of the potential for recall bias or social desirability bias.

## Conclusions

Despite these limitations, our study provides valuable information regarding the actor effect and partner effect of HIV-related stigma on quality of life in dyads consisting PLWHAs and caregivers in China. The findings demonstrate that low quality of life in both PLWHAs and their caregivers and strong association between stigma and quality of life. Because the personal perception of stigma is usually determined by stigmatizing attitudes from outsiders, interventions to reduce HIV-related stigma and improve quality of health should be implemented at individual, family, and community levels. Caregivers of PLWHAs need guidance and assistance, with intervention programs assisting with stress, depression and stigma management and coping strategies to improve quality of life. As HIV stigma and quality of life are complex phenomena rooted in cultures, intervention programs should be carefully planned based on social or cognitive theories and should be culturally adopted.
