# Correction: Associations between Perceived HIV Stigma and Quality of Life at the Dyadic Level: The Actor-Partner Interdependence Model

**DOI:** 10.1371/annotation/2eff8045-349d-4b70-b786-7b85288ffe01

**Published:** 2013-06-14

**Authors:** Hongjie Liu, Yongfang Xu, Xinjin Lin, Jian Shi, Shiyi Chen

The word "Level" is misspelled in the article title. The correct title is: Associations between Perceived HIV Stigma and Quality of Life at the Dyadic Level: The Actor-Partner Interdependence Model. The correct citation is: Liu H, Xu Y, Lin X, Shi J, Chen S (2013) Associations between Perceived HIV Stigma and Quality of Life at the Dyadic Level: The Actor-Partner Interdependence Model. PLoS ONE 8(2): e55680. doi:10.1371/journal.pone.0055680 

